# Supplementary material for: Asymptomatic bacteriuria among pregnant women attending antenatal care at Mbale Hospital, Eastern Uganda
Source: PLoS One. 2020 Mar 19;15(3):e0230523. doi: 10.1371/journal.pone.0230523 (PMC7082119; doi:10.1371/journal.pone.0230523)
Supplement: Attachment — Submitted filename: Reviewers comments.pdf [file pone.0230523.s002.pdf]

Reviewers comments

Asymptomatic bacteriuria among pregnant women attending antenatal care at Mbale hospital, Eastern Uganda.

Its excellent study should be published after some corrections

1. The positive women for asymptomatic is indicated as 22 in the abstract and the results However, the in table 3 there are 28 isolates g-ve 17 and g+ve 11
   Please harmonize this information in the document
2. Page 21 line 5 the statement that some of women could have be taking medicines

   > hygiene all of which increase the risk of developing urinary tract infection (20). It is also possible that some women were using antibiotics at the time of sample collection which could explain the low prevalence in our study as there are a number of privately owned health facilities that have made access to antibiotics easier. If asymptomatic urinary tract infection in pregnancy is not treated,

   This could make more sense if the author could consider calculating the multiple drug resistance index of the microorganisms in the study
