# Supplementary material for: Asymptomatic bacteriuria among pregnant women attending antenatal care at Mbale Hospital, Eastern Uganda
Source: PLoS One. 2020 Mar 19;15(3):e0230523. doi: 10.1371/journal.pone.0230523 (PMC7082119; doi:10.1371/journal.pone.0230523)
Supplement: Attachment — Submitted filename: Response to reviewers.doc [file pone.0230523.s003.doc]

Response to reviewers

A more substantial introduction has been written.

Selection of participants: this has been corrected.

All the women had blood sugar tests carried out on them. None of the women had preexisting or gestational diabetes mellitus.

Mid upper mid circumference was used to calculate the BMI of the women since the women did not know their pre-pregnancy weight. This information has been included in table 1.

In the "Data Collection Procedures" section, further tests were performed for Enterobacteriaceae counts less than 10,000 Cfu/ml, but not for other potential bacterial infections, because Enterobacteriaceae are rarely contaminants whereas other bacteria such as Staphylococcus are contaminants.

In Table 1 “Married” is simple yes or no, because women who were cohabiting were grouped under married, since they only lack a legal marriage.

In table 2, the p-values in the main text for 20-24 age group in comparison to <20 group has been corrected in the text to agree with what is in the table

The typographic error in the title "ANTIICROBIAL SUSCEPTIBILITY OF THE ISOLATES" has been corrected

The date of recruitment has been included.

The sample size calculation: more information has been given about the proportions which were used in the sample size calculation.

Socioeconomic status was assessed using occupational status which has been included in table 1.

The references have been updated.

Phenotypic mechanisms of resistance have been added in the methods section

Ethical approval appears once at the moment.

Bacterial identification and susceptibility testing have been put under a new heading.

A footnote has been added on table 1

Reference 15 is about pregnant women not children.

Other mechanisms of resistance to beta-lactam antibiotics have been included.

The positive women for asymptomatic bacteriuria are reported to be 22 in abstract and in the results. But there were 28 organisms is isolated from the 22 positive samples. This is because some women had duo infection with microorganisms.

Multiple antibiotic resistance indices (MARI) for the microorganisms in the study have been calculated. The method which was used to calculate the indices has been included in the methods section. Table 6 shows the (MARI) for the different microorganisms in the study.
